# Predictive Value of Circulating Tumor Cells in Neoadjuvant Chemoimmunotherapy for Non‐Small Cell Lung Cancer

**DOI:** 10.1111/1759-7714.70288

**Published:** 2026-04-28

**Authors:** Jianyi Yang, Ziwen Qin, Zhiwei Zhou, Jixian Liu, Lin Chen, Ziyan Mo, Mengying Liao, Dongliang Cui, Kaiqin Wu, Dongjiang Tang, Jinfeng Chen, Chao Chen

**Affiliations:** ^1^ Department of Thoracic Surgery Peking University Shenzhen Hospital, Shenzhen Peking University‐The Hong Kong University of Science and Technology Medical Center Shenzhen Guangdong People's Republic of China; ^2^ Department of Thoracic Surgery Peking University Shenzhen Hospital Shenzhen Guangdong People's Republic of China; ^3^ Thoracic Surgery Department Peking University Cancer Hospital & Institute Beijing China; ^4^ Zhuhai Sanmed Biotech Ltd. Zhuhai Guangdong People's Republic of China; ^5^ Department of Pathology Peking University Shenzhen Hospital Shenzhen Guangdong People's Republic of China; ^6^ Shenzhen Key Laboratory of Inflammatory and Immunology Diseases Peking University Shenzhen Hospital Shenzhen Guangdong People's Republic of China

**Keywords:** circulating tumor cells, neoadjuvant chemoimmunotherapy, non‐small cell lung cancer

## Abstract

**Background:**

The incorporation of immune checkpoint blockade into preoperative regimens has significantly advanced the clinical management of locally advanced non‐small cell lung cancer (NSCLC). Standard imaging modalities frequently fall short in assessing actual pathological regression. Our research sought to evaluate circulating tumor cells (CTCs) as an adjunct predictive tool, while uncovering the transcriptomic shifts within the tumor microenvironment that facilitate cellular shedding.

**Methods:**

A prospective cohort of 39 patients with NSCLC received neoadjuvant programmed cell death protein 1 (PD‐1) inhibitors combined with platinum‐doublet chemotherapy. Preoperative radiographic evaluations using RECIST 1.1 were cross‐referenced with final pathological outcomes. Peripheral blood CTCs were enriched and phenotypically characterized via multiparametric immunofluorescence (CK, PD‐L1). Bulk RNA‐sequencing of residual tissue specimens was performed to identify gene signatures associated with CTC dissemination.

**Results:**

The cohort achieved a major pathologic response (MPR) rate of 38.5%, including a pathological complete response (pCR) rate of 23.1%. Preoperative cytokeratin‐positive (CK+) CTC burden emerged as a potential independent predictor of pathological non‐response (AUC = 0.757), outperforming total CTCs and PD‐L1+ CTCs. Crucially, integrating CK+ CTC counts with standard radiographic imaging improved predictive accuracy for pathological outcomes (AUC = 0.79) compared to imaging alone (AUC = 0.54, *p* = 0.022). Transcriptomic profiling of the residual tumor microenvironment suggested that CTC dissemination may be associated with enhanced proliferative activity, severe local hypoxia and a broad immunological suppression within local microenvironments.

**Conclusions:**

Preoperative CTC monitoring may serve as a promising complementary biomarker to conventional radiographic imaging, with the potential to help resolve predictive ambiguities in neoadjuvant chemoimmunotherapy for NSCLC.

## Graphical abstract

Preoperative CK+ CTC monitoring may serve as a promising adjunct to conventional imaging for predicting pathological response in patients receiving neoadjuvant chemoimmunotherapy. Our exploratory findings suggest that systemic CTC dissemination is associated with a microenvironmental convergence of local hypoxia and attenuated immune signaling.

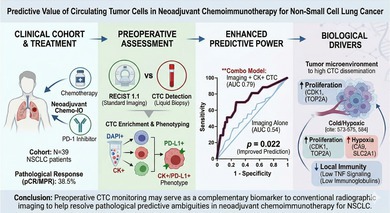

## 1 Introduction

Lung cancer remains the leading cause of cancer‐related mortality worldwide, with non‐small cell lung cancer (NSCLC) accounting for approximately 85% of all diagnosed cases [1]. The principal histological subtypes of NSCLC are lung adenocarcinoma (LUAD) and lung squamous cell carcinoma (LUSC) [2]. Recent years have witnessed a rapid evolution in the management of NSCLC. The advent of immune checkpoint inhibitors (ICIs) has markedly improved clinical outcomes for a subgroup of patients [3]. The incorporation of ICIs into preoperative treatment protocols has fundamentally transformed the therapeutic landscape for patients with borderline resectable NSCLC. Traditional platinum‐based neoadjuvant chemotherapy offers only a modest 5% improvement in 5‐year overall survival, whereas the combination of chemotherapy and immunotherapy has demonstrated superior clinical efficacy [4, 5]. Landmark Phase III trials, such as CheckMate‐816 and KEYNOTE‐671, have reported significantly higher rates of major pathological response (MPR) and pathological complete response (pCR), which are strongly correlated with prolonged event‐free survival (EFS) and overall survival (OS) [6, 7]. Consequently, neoadjuvant chemoimmunotherapy has been widely adopted in clinical settings for stage II–III NSCLC patients without EGFR or ALK alterations, providing a promising therapeutic window for patients lacking targetable driver mutations [5, 8].

While neoadjuvant chemoimmunotherapy has redefined the treatment landscape for NSCLC, its implementation is hindered by several formidable challenges. Although traditional radiographic criteria have proven reliable for monitoring responses to conventional neoadjuvant chemotherapy, their diagnostic utility is notably diminished in the context of neoadjuvant immunotherapy [9]. Conventional imaging techniques struggle to differentiate residual viable tumor cells from therapy‐induced immune infiltration, highlighting the urgent need for complementary predictive biomarkers [10]. The inability to accurately identify high‐response subgroups underscores the urgent need for robust biomarkers, particularly since PD‐L1 expression alone provides insufficient predictive value for pathological response. Furthermore, the phenomenon of primary and acquired resistance remains a significant hurdle, occasionally resulting in treatment failure prior to surgical intervention. Significant debate also surrounds the optimization of perioperative treatment duration. More importantly, the integration of combination therapy is associated with an elevated incidence of treatment‐related adverse events (TRAEs) and severe adverse events (SAEs) compared to monotherapy [11]. Further investigation is imperative to refine the clinical management of neoadjuvant immunotherapy in NSCLC.

Circulating tumor cells (CTCs) are a rare population of cells that exfoliate from primary or metastatic tumor sites and enter the peripheral blood [12]. As a cornerstone of liquid biopsy, the detection and analysis of CTCs offer a non‐invasive, real‐time approach to monitoring tumor dynamics, which overcomes the spatial and temporal limitations of traditional tissue biopsies [13]. Longitudinal CTC monitoring has been rigorously validated as a reliable tool for evaluating therapeutic responses in advanced NSCLC [14, 15]. Crucially, the phenotypic profiling of these circulating populations has established that programmed death‐ligand 1 (PD‐L1) expression on CTCs serves as a robust auxiliary biomarker in metastatic cohorts, simultaneously identifying patients with a poor underlying prognosis and isolating those most likely to derive clinical benefit from systemic immune checkpoint blockade [16, 17]. By logical extension, integrating CTC evaluation into the neoadjuvant phase for locally advanced, resectable NSCLC holds profound theoretical potential for optimizing preoperative risk stratification and guiding surgical timing. However, despite its established clinical utility in advanced‐stage disease, systematic and robust research evidence defining the precise predictive value of CTCs and their PD‐L1 status within the neoadjuvant chemoimmunotherapy setting remains conspicuously lacking.

Driven by this unmet clinical need, we investigated the predictive value of CTC burden and concomitant PD‐L1 expression profiles in a cohort of NSCLC patients undergoing neoadjuvant chemoimmunotherapy. By cross‐referencing these liquid biopsy metrics with standard radiographic assessments and definitive pathological responses, we sought to establish their complementary diagnostic utility for preoperative evaluation. Furthermore, we integrated these clinical outcomes with bulk transcriptomic profiling of the residual tumor microenvironment to elucidate the biological drivers of CTC dissemination. This multidimensional approach aims to provide a robust theoretical framework to refine efficacy assessments, resolve imaging ambiguities, and optimize individualized surgical and neoadjuvant adjuvant therapeutic strategies.

## 2 Methods

### 2.1 Patients

**FIGURE 1:**
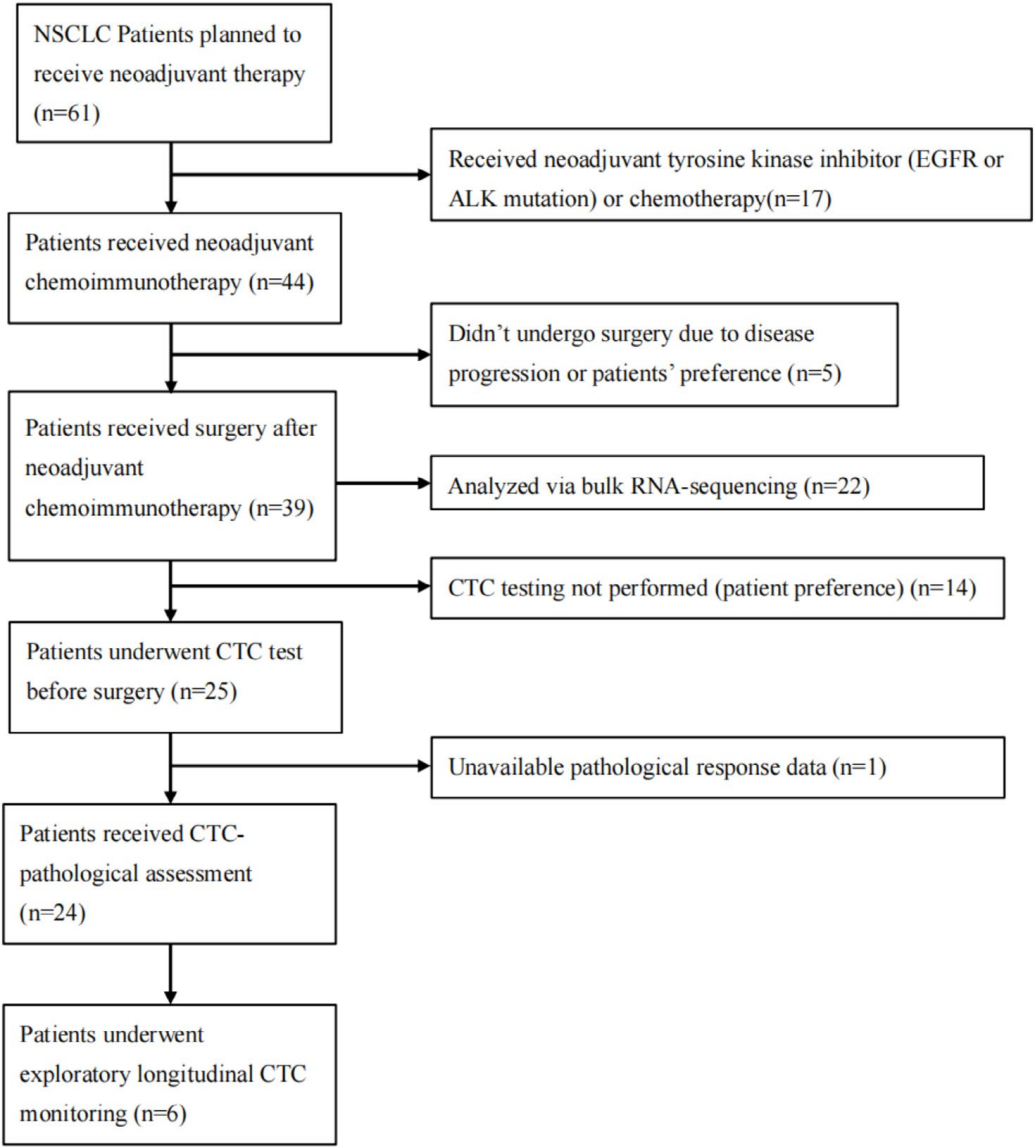
Study flow diagram illustrating patient enrollment and sub‐analysis stratification. A total of 1462 patients scheduled for lung cancer surgery were screened. Following the exclusion of patients who underwent direct surgery (*n* = 1401) or received non‐immuno‐neoadjuvant regimens (*n* = 17), 44 patients received neoadjuvant chemoimmunotherapy. Five patients were excluded due to disease progression or personal preference, resulting in a final surgical cohort of 39 patients. Sub‐analyses were performed based on specimen availability for preoperative CTC testing (*n* = 25), bulk RNA‐sequencing (*n* = 22), and exploratory longitudinal monitoring (*n* = 6).

A total of 61 NSCLC patients scheduled for neoadjuvant therapy at Peking University Shenzhen Hospital between December 2024 and December 2025 were initially screened (Figure 1). As detailed in the study flow diagram, patients who received neoadjuvant tyrosine kinase inhibitors or chemotherapy alone (*n* = 17) were excluded. Of the 44 patients who initiated neoadjuvant chemoimmunotherapy, 5 patients did not proceed to surgical intervention due to disease progression or personal preference. The final cohort consisted of 39 patients with histologically confirmed NSCLC who underwent radical resection following neoadjuvant chemoimmunotherapy. Patients were enrolled based on strictly defined criteria. The primary inclusion criteria were: (1) Pathologically confirmed primary NSCLC; (2) Age between 18 and 80; (3) Clinical staging appropriate for neoadjuvant or conversion therapy (generally encompassing resectable, borderline resectable, or potentially resectable advanced disease); (4) An Eastern Cooperative Oncology Group (ECOG) performance status score of 0 or 1; (5) Adequate bone marrow, hepatic, and renal organ function. Exclusion criteria were as follows: (1) Previous systemic anti‐tumor treatment or radiotherapy; (2) Previous other malignancies; (3) Presence of sensitive driver gene mutations in patients with adenocarcinoma (specifically confirmed as pan‐negative for *EGFR* or *ALK* alterations); (4) Active autoimmune diseases, immunodeficiency, or systemic immunosuppressive therapy requiring systemic corticosteroids.

### 2.2 Treatment Protocol

Prior to treatment initiation, all patients underwent standard staging examinations including contrast‐enhanced computed tomography (CT), magnetic resonance imaging (MRI) of the brain, bone scintigraphy, and ultrasonography (liver, adrenal glands, kidneys, supraclavicular and cervical lymph nodes), and/or positron emission tomography (PET)‐CT. All therapeutic strategies were formulated following comprehensive evaluation by a multidisciplinary team (MDT).

All enrolled patients received standard neoadjuvant or conversion therapy consisting of a programmed cell death protein 1 (PD‐1) inhibitor combined with platinum‐based doublet chemotherapy. The specific chemotherapeutic regimen was tailored to the histological subtype: patients with squamous cell carcinoma routinely received paclitaxel plus a platinum agent, whereas those with pan‐negative adenocarcinoma received pemetrexed plus a platinum agent.

The planned neoadjuvant treatment phase typically consisted of 2 to 4 cycles. A subset of patients presenting with initially unresectable Stage IIIb–IV disease received this combination regimen with the intent of conversion therapy. The definitive number of preoperative cycles and the optimal timing for surgical intervention were individualized for each patient, determined by sequential MDT discussions that integrated radiological response, drug tolerance, and overall surgical feasibility. Detailed baseline clinical characteristics and individual treatment regimens are provided in Table S1.

Following the completion of neoadjuvant therapy, all eligible patients underwent either video‐assisted thoracoscopic surgery (VATS) or open radical resection. These procedures systematically included lobectomy or bilobectomy accompanied by comprehensive hilar and mediastinal lymph node dissection in accordance with standard oncological principles. The study protocol for this study and all procedures involving human participants were approved by the Ethics Committee of Peking University Shenzhen Hospital (No. 2020‐074) and informed consent was obtained from all individual participants.

### 2.3 Imaging and Histopathological Evaluation Criteria

Radiographic efficacy was assessed by comparing baseline and preoperative contrast‐enhanced computed tomography (CT) or PET‐CT scans to ensure consistent measurement of tumor burden. Two independent, blinded physicians evaluated the objective therapeutic response strictly in accordance with the Response Evaluation Criteria in Solid Tumors (RECIST) version 1.1 guidelines. Based on the changes in the sum of the longest diameters of target lesions, patients were categorized into complete response (CR), partial response (PR), stable disease (SD), or progressive disease (PD) to guide subsequent surgical planning.

All excised tissue specimens were systematically sectioned and independently reviewed by two experienced thoracic pathologists who were blinded to the patients' clinical and radiographic treatment responses. Pathological efficacy was quantitatively determined by estimating the percentage of residual viable tumor (RVT) cells within the cross‐sectional area of the primary tumor bed using standard hematoxylin and eosin (H&E) staining. Specifically, a major pathological response (MPR) was defined as the presence of 10% or less RVT, while a pathological complete response (pCR) was established when there was a complete absence of viable tumor cells in both the primary lesion and all resected lymph nodes.

### 2.4 CTC Isolation, Detection, and Characterization

In this study, Cytokeratin (CK) was selected as the primary epithelial marker for CTC identification, as CKs (including CK7, CK8, CK18, and CK19) are intermediate filament proteins widely expressed in epithelial‐derived malignancies, including non‐small cell lung cancer [18]. The use of pan‐CK antibodies in combination with CD45 exclusion enables specific discrimination of CTCs from background leukocytes. While CK‐based detection is well‐established in platforms such as CellSearch, we acknowledge that CTCs undergoing epithelial–mesenchymal transition may downregulate CK expression, potentially leading to underdetection. PD‐L1 was selected as the second phenotypic marker given its established role as a predictive biomarker for immune checkpoint inhibitor therapy [19].

Approximately 10 mL of peripheral blood was collected from each patient within 1 week before operation. Peripheral blood mononuclear cells (PBMCs) were subsequently isolated via density gradient centrifugation. The collected PBMCs were fixed in 1% paraformaldehyde (PFA) for 1 h at room temperature and washed twice with binding buffer.

The fixed cell suspensions were incubated with 20 μL of a mixed capture reagent (Zhuhai Sanmed Biotech Ltd., Zhuhai, China), supplemented with 8 μL of anti‐cytokeratin (CK), 5 μL of anti‐PD‐L1, and 10 μL of anti‐CD45 detection antibodies overnight at 2°C–8°C. Following two washes with binding buffer, the cells were incubated with 30 μL of avidin magnetic beads and 5 μL of a fluorescent secondary antibody (Anti‐HRP AF594; Jackson ImmunoResearch) for 1 h at 2°C–8°C in the dark.

Automated CTC enrichment and isolation were performed using the LiquidBiopsy Rare Cell Isolation System (LIQUIDBIOPSY400A; Zhuhai Sanmed Biotech Ltd.). The enriched cells were counterstained with 4′,6‐diamidino‐2‐phenylindole (DAPI) for nuclear visualization and subsequently scanned and analyzed using an automated fluorescence microscope (Leica DM6000B; Leica Microsystems). CTCs were strictly defined as morphologically intact cells exhibiting DAPI+/CK+/PD‐L1+/CD45‐, DAPI+/CK+/PD‐L1‐/CD45‐, or DAPI+/CK‐/PD‐L1+/CD45‐ phenotypes.

### 2.5 RNA Sequencing

1. Tissue Collection and RNA Extraction. Viable tumor tissues were harvested from active growth margins within 30 min of surgical resection, with strict exclusion of necrotic or hemorrhagic regions. Tissue fragments (approximately 3 × 3 × 3 mm) were immediately stabilized in RNAlater solution (Invitrogen, Carlsbad, CA, USA) overnight at 4°C and subsequently cryopreserved at −80°C. Total RNA was isolated from tissue and cell samples utilizing TRIzol reagent (Invitrogen) according to standard manufacturer guidelines. RNA integrity and concentration were rigorously assessed using a Fragment Analyzer, an Agilent 2100 Bioanalyzer (Agilent Technologies, CA, USA), or a Qseq‐400 system (Bioptic, Taiwan, China).
2. Library Construction and RNA Sequencing. Transcriptomic libraries were prepared using the BGI Optimal Dual‐Module mRNA Library Preparation Kit (BGI‐Shenzhen, China). Briefly, poly(A) mRNA was enriched via oligo(dT) magnetic beads, fragmented, and reverse‐transcribed into double‐stranded cDNA. Following end repair, 3′ adenylation, and sequencing adapter ligation, the amplified PCR products were circularized to generate single‐stranded circular DNA. These constructs underwent rolling circle amplification (RCA) to form DNA nanoballs (DNBs). Sequencing was executed on the BGI DNBSEQ platform (G400, T7, or T10) utilizing combinatorial Probe‐Anchor Synthesis (cPAS) technology with a paired‐end 100/150 bp (PE100/150) strategy.

### 2.6 Bioinformatics Analysis

Raw gene count matrices derived from bulk RNA sequencing were analyzed using the R software environment. To assess the transcriptomic differences associated with tumor dissemination, patients were dichotomized based on their preoperative CTC status (CTC‐positive vs. CTC‐negative). Differential gene expression analysis was conducted using the *DESeq2* R package. To reduce statistical noise and improve the power of multiple testing correction, low‐expressed genes were rigorously pre‐filtered; only genes with read counts ≥ 10 in at least two samples were retained for downstream analysis. The Wald test was utilized to calculate *p*‐values, which were subsequently adjusted using the Benjamini–Hochberg procedure to control the false discovery rate (FDR). Differentially expressed genes (DEGs) were robustly defined by an adjusted *p*‐value (*p*adj) < 0.05 and an absolute log2 fold change (|log2FC|) > 1.0.

To elucidate the broader biological pathways associated with CTC dissemination, Gene Set Enrichment Analysis (GSEA) was performed using the *clusterProfiler* R package. All detected genes were ranked by their log2 fold change values, and this pre‐ranked gene list was analyzed against Gene Ontology (GO) and Kyoto Encyclopedia of Genes and Genomes (KEGG) pathway databases using the gseGO and gseKEGG functions, respectively. Pathways with a Benjamini–Hochberg adjusted *p*‐value < 0.05 were considered significantly enriched. Activated pathways (normalized enrichment score [NES] > 0) represent gene sets enriched in CTC‐positive tumors, while suppressed pathways (NES < 0) represent gene sets enriched in CTC‐negative tumors. Unsupervised hierarchical clustering of the top 30 DEGs was performed using the pheatmap R package with Ward.D2 linkage and Euclidean distance, and expression values were *z*‐score normalized across samples. Data visualizations, including volcano plots, GSEA enrichment dot plots, and clustered heatmaps, were generated using the ggplot2, ggrepel, enrichplot, and pheatmap packages.

### 2.7 Statistical Analysis

Continuous variables were summarized as medians with interquartile ranges (IQRs), and categorical variables were presented as frequencies and percentages. To evaluate the relationship between circulating biomarkers and pathological outcomes, patients were dichotomized into two primary cohorts: the MPR group, which encompasses all individuals achieving either a major pathological response or a pathological complete response, and the NMPR (non‐major pathological response) group, comprising those with more than 10% residual viable tumor. Given the biological premise that lower preoperative CTC burdens correlate with favorable treatment responses, the comparisons of continuous preoperative CTC counts (Total, CK+, and PD‐L1+) between the MPR and NMPR cohorts were performed using a one‐sided Welch's *t*‐test. For the purpose of continuous variable modeling, patients with no detectable CTCs were assigned a numerical value of zero for all subtypes. The spatial concordance between tissue PD‐L1 expression (TPS ≥ 1%) and CTC PD‐L1 status (≥ 1 cell) was assessed by calculating the overall agreement rate.

To estimate the risk of pathological non‐response, univariate logistic regression models were initially utilized to calculate odds ratios (ORs) and 95% confidence intervals (CIs) for both clinical covariates and specific CTC subtypes. To rigorously evaluate the independent predictive value of CTCs while accounting for potential baseline confounders (e.g., clinical stage), multivariate logistic regression analysis was subsequently performed. The diagnostic performance of individual biomarkers, radiological evaluation alone, and the combination models (Radiological Evaluation + CTC metrics) was evaluated using receiver operating characteristic (ROC) curves, and the area under the curve (AUC) and 95% Confidence Interval (CI) was calculated. The incremental predictive value of adding CTC counts to the preoperative radiological evaluation was rigorously tested by comparing the AUCs using DeLong's test. All statistical analyses and visualizations were performed using R software (version 4.x). Unless otherwise specified (e.g., the prespecified one‐sided *t*‐test), a two‐sided *p*‐value < 0.05 was considered statistically significant.

## 3 Results

### 3.1 Baseline Characteristics of Patients

A total of 39 NSCLC patients who received neoadjuvant chemoimmunotherapy were enrolled in this study (Figure 1). The baseline clinical and demographic characteristics of the cohort were summarized in Tables 1 and S1. The median age of the patients was 66 years old (60–70), and the cohort was predominantly male (94.9%, 37/39). Smoking status was relatively balanced, with 48.7% categorized as never‐smokers and 51.3% as former or current smokers. Regarding histological subtypes, LUSC was the most common, accounting for 66.7% (26/39) of the patients, followed by LUAD in 28.2% (11/39). At initial diagnosis, over half of the cohort (56.4%) presented with Stage III–IV disease, while 43.6% had stage I–II disease. A total of 22 patients received two cycles of neoadjuvant treatment, whereas 17 patients received more than two cycles. A major pathological response (MPR) rate of 38.5% (15/39), including a pathological complete response (pCR) rate of 23.1% (9/39) observed in this cohort demonstrates robust consistency with the benchmark efficacy outcomes established by CheckMate‐816 (MPR: 36.9%, pCR: 24.0%) [6] and KEYNOTE‐671 (MPR: 30.2%, pCR: 18.1%) [7].

**TABLE 1:** Patients' characteristic.

Characteristics	Total cohort (*N* = 39)	MPR group (*N* = 15)	NMPR group (*N* = 23)
Age, median [IQR], years	66 [60–70]	66 [61–70]	64 [58–69]
Sex, *n* (%)			
Male	37 (94.9%)	15 (100.0%)	21 (91.3%)
Female	2 (5.1%)	0 (0.0%)	2 (8.7%)
Smoking status, *n* (%)			
Never	19 (48.7%)	8 (53.3%)	11 (47.8%)
Former/Current	20 (51.3%)	7 (46.7%)	12 (52.2%)
Histology, *n* (%)			
LUSC	26 (66.7%)	12 (80.0%)	13 (56.5%)
LUAD	11 (28.2%)	3 (20.0%)	8 (34.8%)
Others	2 (5.1%)	0 (0.0%)	2 (8.7%)
Clinical Stage, *n* (%)			
Stage I–II	17 (43.6%)	9 (60.0%)	7 (30.4%)
Stage III–IV	22 (56.4%)	6 (40.0%)	16 (69.6%)
Neoadjuvant Cycles, *n* (%)			
≤ 2 cycles	22 (56.4%)	6 (40.0%)	15 (65.2%)
> 2 cycles	17 (43.6%)	9 (60.0%)	8 (34.8%)
Radiological Response, *n* (%)			
CR (Complete Response)	1 (2.6%)	1 (6.7%)	0 (0.0%)
PR (Partial Response)	22 (56.4%)	11 (73.3%)	10 (43.5%)
SD (Stable Disease)	15 (38.5%)	3 (20.0%)	12 (52.2%)
PD (Progressive Disease)	1 (2.6%)	0 (0.0%)	1 (4.3%)
Tissue PD‐L1 Expression, *n* (%)			
Negative (< 1%)	3 (7.7%)	0 (0.0%)	3 (13.0%)
Low (1%–49%)	8 (20.5%)	4 (26.7%)	4 (17.4%)
High (≥ 50%)	7 (17.9%)	6 (40.0%)	1 (4.3%)
Not Evaluated (NA)	21 (53.8%)	5 (33.3%)	15 (65.2%)
Preoperative Total CTCs, *n* (%)			
Negative (0)	13 (33.3%)	8 (53.3%)	4 (17.4%)
Positive (≥ 1)	12 (30.8%)	4 (26.7%)	8 (34.8%)
Not Evaluated (NA)	14 (35.9%)	3 (20.0%)	11 (47.8%)
Preoperative CK+ CTCs, *n* (%)			
Negative (0)	14 (35.9%)	10 (66.7%)	4 (17.4%)
Positive (≥ 1)	11 (28.2%)	2 (13.3%)	8 (34.8%)
Not Evaluated (NA)	14 (35.9%)	3 (20.0%)	11 (47.8%)
Preoperative PD‐L1+ CTCs, *n* (%)			
Negative (0)	16 (41.0%)	9 (60.0%)	7 (30.4%)
Positive (≥ 1)	9 (23.1%)	3 (20.0%)	5 (21.7%)
Not Evaluated (NA)	14 (35.9%)	3 (20.0%)	11 (47.8%)
RNA‐seq Performed, *n* (%)			
Yes	22 (56.4%)	11 (73.3%)	11 (47.8%)
No	17 (43.6%)	4 (26.7%)	12 (52.2%)

### 3.2 CTC and CTC CK Predict Pathological Response to Neoadjuvant Immunotherapy

**FIGURE 2:**
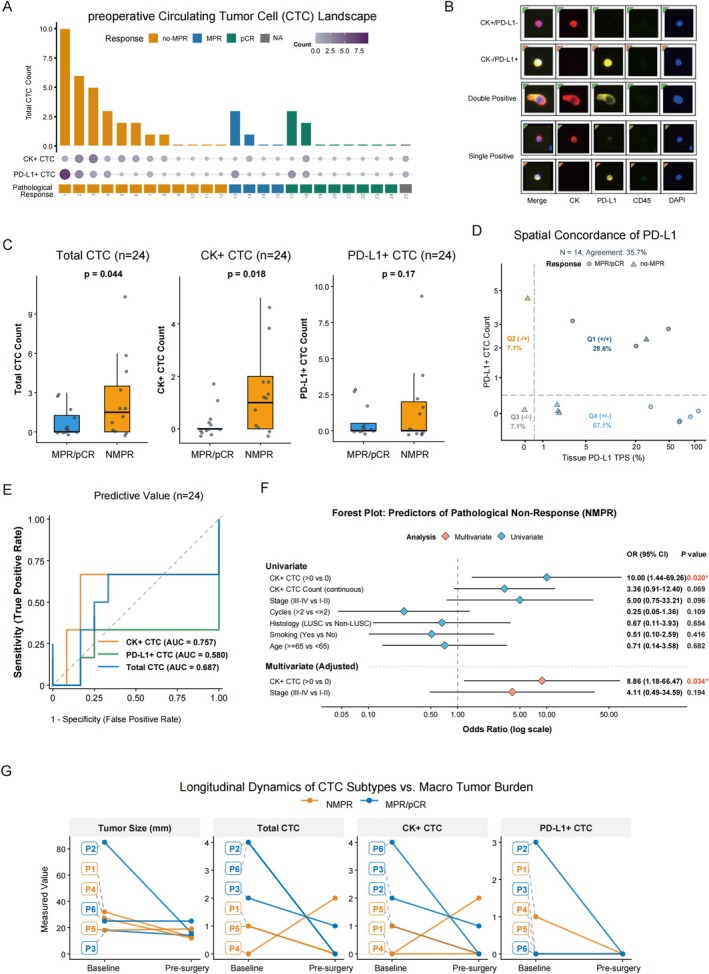
Predictive value of CTCs in patients receiving neoadjuvant chemoimmunotherapy. (A) The preoperative CTC landscape across the evaluated patient cohort. The upper bar chart displays the total CTC count for each patient, aligned with corresponding dot plots indicating the detection of specific phenotypic subsets (CK+ CTCs and PD‐L1+ CTCs). Patients are ordered by total CTC burden and color‐coded according to their definitive pathological response (no‐MPR, MPR, pCR, or NA) (B) Representative multiparametric immunofluorescence images of the enriched CTCs. Cells were counterstained with DAPI (nucleus, blue) and evaluated for cytokeratin (CK, red), programmed death‐ligand 1 (PD‐L1, yellow), and CD45 (leukocyte exclusion marker, purple). Distinct phenotypic subpopulations are shown, including CK+/PD‐L1‐ (single positive), CK‐/PD‐L1+ (single positive), and CK+/PD‐L1+ (double positive) cells. (C) Box plots comparing the preoperative continuous counts of Total CTCs, CK+ CTCs, and PD‐L1+ CTCs between the MPR/pCR and NMPR cohorts (*n* = 24). (D) Spatial concordance analysis comparing baseline primary tissue PD‐L1 tumor proportion score (TPS, %) with the corresponding preoperative peripheral blood PD‐L1+ CTC count (*n* = 14). The dashed lines establish the categorical positivity thresholds, with the overall agreement rate denoted. (E) Receiver operating characteristic (ROC) curves evaluating the diagnostic performance of preoperative Total CTCs, CK+ CTCs, and PD‐L1+ CTCs for predicting pathological response. The area under the curve (AUC) is provided for each biomarker. (F) Forest plot illustrating the predictors of pathological non‐response (NMPR) using logistic regression models. (G) Longitudinal kinetic trajectories of macroscopic tumor size (mm) alongside Total, CK+, and PD‐L1+ CTC counts. Data points capture the transition from preoperative to the pre‐surgery timepoint for an exploratory subset of patients, with individual trajectories color‐coded by their final pathological outcome (MPR/pCR vs. NMPR).

A total of 25 patients underwent preoperative CTC assessment (Figure 2A). The maximum number of CTCs detected was 10, while 12 patients had detectable CTCs. Among these, the CTC positivity rate was 66.7% (8/12) in the non‐major pathological response (NMPR) group, compared to 33.3% (4/12) in the MPR group (including pCR). One patient was excluded from subsequent analyses due to unavailable pathological response data. Those without detectable CTCs were assigned a count of zero for all phenotypic subtypes (CK+ or PD‐L1+) for the purpose of logistic regression and ROC modeling. Of note, two patients (pt 17 and pt 18) had detectable CTCs in preoperative blood samples despite achieving pCR in surgical specimens, suggesting a potential risk of occult residual disease. As of the latest follow‐up (3.3 and 9.4 months, respectively), neither patient showed evidence of recurrence. Preoperative evaluations revealed that 48%, 44%, and 36% of the cohort tested positive for total CTCs, CK+ CTCs, and PD‐L1+ CTCs, respectively. These phenotypic subsets were successfully identified and differentiated into single or double‐positive populations using multiparametric immunofluorescence (CK, PD‐L1, CD45, DAPI) (Figure 2B). Quantitative analysis of this landscape revealed that patients in the non‐major pathological response (NMPR) group harbored significantly higher preoperative counts of both total CTCs (*p* = 0.044) and CK+ CTCs (*p* = 0.018) compared to those achieving an MPR or pCR (Figure 2C). Conversely, preoperative PD‐L1+ CTC counts did not demonstrate a statistically significant difference between the two response groups (*p* = 0.17) (Figure 2C). Among the cohort, 14 patients had paired assessments for treatment‐naïve tissue PD‐L1 tumor proportion score (TPS) and post‐treatment CTC PD‐L1 expression (Figure 2D). A comparative analysis revealed a low spatial concordance rate of 35.7% (5/14) between the pre‐treatment tissue specimens and the post‐treatment CTC samples. Notably, baseline tissue PD‐L1 expression was highly indicative of clinical benefit; patients harboring a baseline tumor biopsy PD‐L1 TPS ≥ 5% demonstrated a markedly higher MPR rate compared to those below this threshold (85.7% vs. 14.3%, respectively).

Receiver operating characteristic (ROC) analysis showed that preoperative CK+ CTCs had the best predictive performance for pathological response in this cohort (AUC = 0.757 (95% CI: 0.578–0.936)), with a higher AUC value than total CTCs (AUC = 0.687 (95% CI: 0.483–0.892)) and PD‐L1+ CTCs (AUC = 0.580 (95% CI: 0.381–0.779)) (Figure 2E). To rigorously validate whether CK+ CTCs serve as an independent predictor of treatment efficacy, we constructed univariate and multivariate logistic regression models (Figure 2F). In the univariate analysis, the presence of CK+ CTCs (> 0) was strongly associated with a higher risk of NMPR in this cohort (OR = 10.00, 95% CI: 1.44–69.26, *p* = 0.020). To account for baseline clinical confounders, we proceeded with a multivariate regression adjusted for clinical stage (Stage III–IV vs. I–II). Notably, even after adjustment, the preoperative presence of CK+ CTCs retained its statistical significance as an independent predictor of pathological non‐response in this cohort (Adjusted OR = 8.86, 95% CI: 1.18–66.47, *p* = 0.034).

A total of six patients underwent longitudinal CTC monitoring before and after neoadjuvant immunotherapy. The integrated analysis of CTC dynamics, radiographic tumor regression, and pathological response is displayed (Figure 2G). At the individual level, Patient 2 demonstrated substantial radiographic tumor regression accompanied by the complete clearance of CTCs, which aligned with a final MPR outcome. In contrast, Patient 4 exhibited marked radiographic shrinkage alongside a paradoxical increase in CTC counts, ultimately resulting in an NMPR. Notably, despite showing minimal radiographic changes, Patients 3 and 6 displayed declining CTC trajectories and successfully achieved an MPR/pCR. Collectively, these exploratory individual kinetic profiles suggested that longitudinal CTC dynamics may complement conventional radiographic assessments in predicting true pathological response.

### 3.3 Combining Radiographic Imaging With CTC Analysis Enhances the Prediction of Pathological Response Following Neoadjuvant Immunotherapy

**FIGURE 3:**
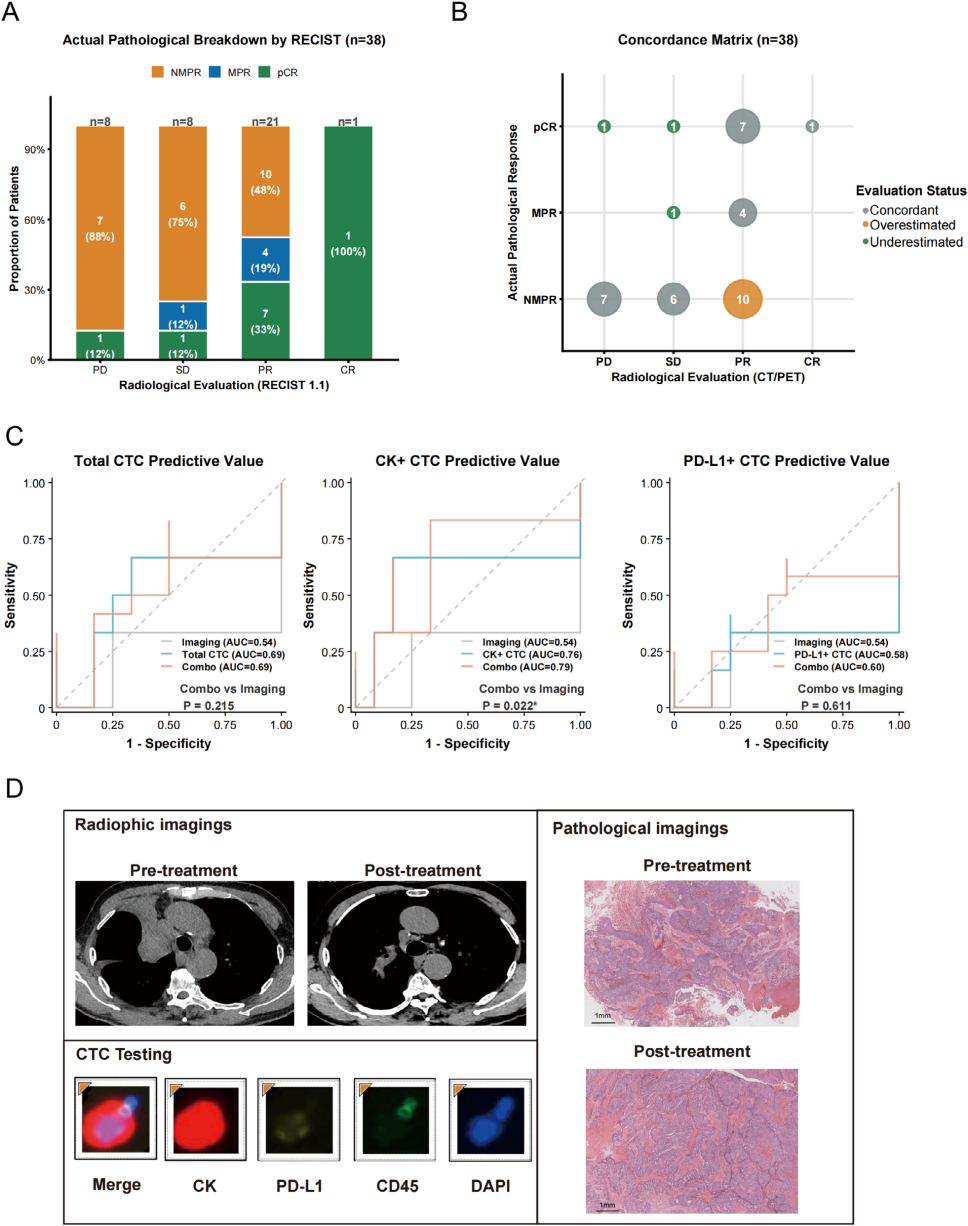
Radiographic‐pathological discordance and predictive value of integrated CTC‐imaging models. (A) Proportional distribution of pathological outcomes (NMPR, MPR, pCR) stratified by preoperative RECIST 1.1 radiological assessments (*n* = 38). (B) Concordance matrix mapping the diagnostic alignment between radiological evaluation and actual pathological clearance. Bubble size indicates patient count; colors denote whether radiological assessments were concordant, overestimated, or underestimated compared to final pathology. (C) ROC curves comparing the diagnostic performance of imaging alone (AUC = 0.54) versus integrated models (Combo) incorporating Total CTCs, CK+ CTCs, or PD‐L1+ CTCs. Statistical comparison via DeLong's test demonstrates a significant predictive enhancement exclusively for the CK+ CTC combination model (*p* = 0.022). (D) Clinical, pathological, and CTC testing of a representative patient. Radiographic evaluation shows PR after treatment and peripheral blood captured one CTC with the phenotype CK+/PD‐L1‐/DAPI+/CD45‐. While pathology showed NMPR.

To further investigate the predictive accuracy of standard radiographic assessments and evaluate the added clinical value of liquid biopsy, we cross‐referenced preoperative radiological evaluations (RECIST 1.1) with the final actual pathological responses (*n* = 38). As illustrated in Figure 3A, the probability of achieving a pCR or MPR generally improved as the degree of radiographic response increased. Patients situated at the extremes of radiographic evaluation demonstrated a high degree of predictive certainty; for instance, the patient achieving a radiographic CR was confirmed to have a pCR pathologically. However, for most of the cohort exhibiting either a PR or SD, morphological imaging demonstrated variable predictive concordance. Specifically, among patients radiologically classified as PR, nearly half (48%) were pathologically confirmed as NMPR, indicating an overestimation of therapeutic efficacy by standard CT criteria. Conversely, conventional imaging also underestimated tumor clearance in a subset of patients, as 24% of those with SD paradoxically achieved either a pCR (12%) or an MPR (12%). Of particular note, one patient assessed with progressive disease (PD) on CT imaging was ultimately confirmed to have achieved a pCR upon surgical resection, representing a clear, clinically significant case of immune‐mediated pseudoprogression. A concordance matrix visually mapped this relationship, explicitly highlighting the distinct clusters where standard imaging either overestimated or underestimated the true pathological outcome (Figure 3B).

We subsequently investigated the integration of preoperative CTC metrics with radiographic imaging data to determine whether preoperative predictive accuracy could be further optimized (Figure 3C). Receiver operating characteristic (ROC) analysis revealed that radiographic imaging alone yielded an area under the curve (AUC) of 0.54 (95% CI: 0.353–0.731) for predicting pathological response. Crucially, integrating CK+ CTC counts with imaging data (Combo) improved predictive performance, elevating the AUC to 0.79 (95% CI: 0.578–0.936) (*p* = 0.022 versus imaging alone). In contrast, combining imaging with either Total CTCs (AUC = 0.69 (95% CI: 0.477–0.905), *p* = 0.215) or PD‐L1+ CTCs (AUC = 0.60 (95% CI: 0.372–0.823), *p* = 0.611) did not yield a statistically significant improvement over radiographic assessment alone. Collectively, these exploratory data suggested that complementing traditional CT evaluation specifically with CK+ CTC monitoring may enhance the preoperative prediction of pathological response in neoadjuvant immunotherapy. Figure 3D illustrates a representative patient who achieved significant radiographic tumor response on CT after neoadjuvant therapy. However, one CK+ PD‐L1‐ CTC was detected preoperatively. Pathological examination of the surgical specimen ultimately revealed massive residual tumor.

### 3.4 Transcriptomic Profiling Reveals Distinct Tumor Microenvironment Features Associated With CTC Positivity

**FIGURE 4:**
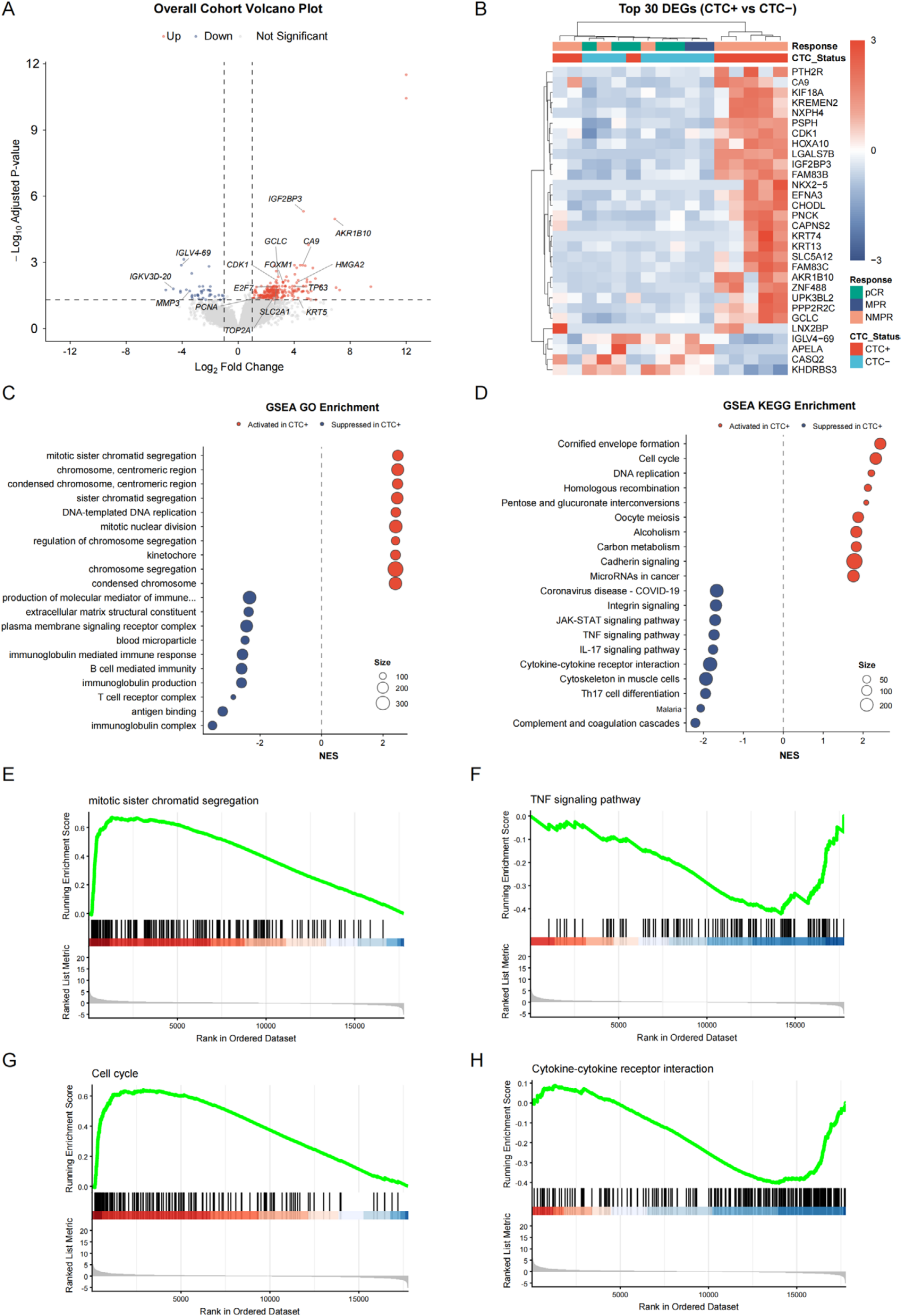
Microenvironmental transcriptomic profiling and functional enrichment analysis stratified by CTC status. (A) Volcano plot of differentially expressed genes (DEGs) between CTC+ and CTC‐ residual tumors. Red and blue dots denote significantly up‐ and down‐regulated genes, respectively (|log_2_ FC| > 1.0, adjusted *p* < 0.05). (B) Unsupervised hierarchical clustering heatmap of the top 30 DEGs across the sequenced cohort, annotated by pathological response and CTC status. (C, D) GO (C) and KEGG (D) pathway enrichment analyses. Red (NES > 0) and blue (NES < 0) bubbles indicate activated and suppressed pathways in CTC+ tumors, respectively. (E–H) Representative GSEA plots for key altered biological networks in CTC+ patients, including activated pathways: Mitotic sister chromatid segregation (E) and Cell cycle (G); and suppressed pathways: TNF signaling pathway (F) and Cytokine‐cytokine receptor interaction (H). CTC, circulating tumor cell; DEGs, differentially expressed genes; GSEA, gene set enrichment analysis; MPR, major pathological response; NES, normalized enrichment score; NMPR, non‐major pathological response; pCR, pathological complete response.

To explore the molecular and microenvironmental features associated with tumor dissemination, we performed bulk RNA‐sequencing on residual tissue specimens, comparing the transcriptomic profiles between CTC‐positive and CTC‐negative patients. The overall cohort volcano plot (Figure 4A) and the heatmap of the top 30 differentially expressed genes (DEGs) (Figure 4B) revealed distinct transcriptomic patterns distinguishing the two groups. Tumors from CTC‐positive patients exhibited significant upregulation of proliferation‐ and cell cycle‐related genes (e.g., *CDK1*, *TOP2A*, *PCNA*, *FOXM1*, *E2F7*), alongside key hypoxia and metabolic reprogramming markers (*CA9*, *SLC2A1*). Conversely, immunoglobulin‐related transcripts, such as *IGLV4‐69* and *IGKV3D‐20*, were markedly downregulated in these patients.

Consistent with individual gene alterations, GSEA of the KEGG and GO databases demonstrated that CTC‐positive tumors were enriched for cell cycle regulation, DNA replication, and mitotic pathways (Figure 4C,D). Specifically, pathways such as “mitotic sister chromatid segregation” (Figure 4E) and “Cell cycle” (Figure 4G) were significantly activated. These findings suggest a higher endogenous proliferative activity within the local tumor lesions that may be associated with peripheral CTC shedding.

Conversely, GSEA revealed a profound suppression of immune‐related networks in the residual tumors of CTC‐positive patients. GO enrichment indicated an attenuation of local humoral immune signals, characterized by the suppression of “immunoglobulin mediated immune response,” “B cell mediated immunity,” and “immunoglobulin production” (Figure 4C). Furthermore, KEGG enrichment highlighted the widespread downregulation of critical inflammatory and immune‐signaling cascades, including the TNF signaling pathway (Figure 4F), IL‐17 signaling, Th17 cell differentiation, and cytokine‐cytokine receptor interactions (Figure 4D,H).

Collectively, these exploratory findings provide clues that a profound suppression of both humoral and cellular immunity within the tumor microenvironment, acting in concert with local hypoxia and enhanced proliferative features, may facilitate CTC dissemination during neoadjuvant chemoimmunotherapy.

## 4 Discussion

For patients with locally advanced or borderline resectable NSCLC, neoadjuvant chemoimmunotherapy has emerged as a standard perioperative treatment strategy. For this subgroup of patients, accurate patient selection and rigorous therapeutic response monitoring are critical to optimally coordinate neoadjuvant systemic treatment with subsequent surgical resection, thereby maximizing durable clinical benefit and improving long‐term oncological outcomes. Despite its widespread use as the standard imaging modality, RECIST‐based CT imaging suffers from critical limitations: it cannot reliably differentiate viable tumor tissue from post‐treatment inflammatory infiltration or fibrosis, thereby leading to inaccurate and delayed evaluation of pathological response. Accordingly, the unmet clinical need for reliable non‐invasive preoperative biomarkers to predict treatment benefit has brought CTCs into the spotlight as a promising candidate in this setting. The present exploratory study revealed the potential predictive value of preoperative CTC testing for assessing pathological response in patients receiving neoadjuvant chemoimmunotherapy. Crucially, we found that integrating CTC testing with standard radiographic imaging significantly optimizes efficacy evaluations, overcoming the limitations of morphological assessment and guiding optimal surgical timing. Furthermore, exploratory transcriptomic profiling suggests that profound suppression of both humoral and cellular immunity within the tumor microenvironment, acting in concert with local hypoxia and enhanced proliferative activity, may provide the biological conditions that facilitate CTC dissemination during neoadjuvant immunotherapy.

In our cohort, a notable discordance was observed between radiological evaluation and actual pathological response. While patients exhibiting a PR on conventional radiological imaging did indeed demonstrate a high rate of MPR post‐surgery, radiological prediction was not entirely accurate, as it failed to capture the full spectrum of pathological clearance in several cases. Conventional CT imaging often mischaracterizes responses to ICIs, as standard size‐based assessments are frequently confounded by pseudoprogression—a transient enlargement driven by immune infiltration, edema, and necrosis [20]. To mitigate this, immune‐related response criteria, notably iRECIST, were introduced to help clinicians differentiate true progression from pseudoprogression [21]. However, recent systematic reviews and meta‐analyses indicate that while iRECIST effectively identifies pseudoprogression in a small subset of patients (approximately 4%–6%), its overall statistical impact on improving objective response rates (ORR) and disease control rates (DCR) remains marginal when compared to traditional RECIST 1.1 [9, 22]. In the specific setting of neoadjuvant chemoimmunotherapy for NSCLC, this radiological‐pathological “disconnect” is even more pronounced. Recent literature confirms that standard CT‐based size measurements are often unreliable predictors of MPR or pathological complete response (pCR) prior to surgical resection [23]. Because neoadjuvant immunotherapy induces profound inflammatory responses and tissue remodeling within the tumor microenvironment, lesions categorized as stable disease (SD) on CT may harbor no viable tumor cells upon pathological examination. To overcome the inherent limitations of conventional CT evaluations, contemporary research is actively pivoting toward advanced multimodal imaging techniques—such as CT‐based radiomics [23]. Dual‐phase CT artificial intelligence models [24], and dynamic 18F‐FDG PET/CT metabolic parameters [25]—to better forecast pathological response. Against this backdrop, our findings highlight the potential clinical utility of CTC dynamic monitoring as a liquid‐based adjunct to conventional imaging for the early and precise prediction of pathological clearance.

Our findings suggested that preoperative CTC burden may serve as a potential independent predictive biomarker for pathological response in NSCLC patients receiving neoadjuvant chemoimmunotherapy. As a cornerstone of liquid biopsy, CTCs—intact cancer cells shed from the primary tumor into the peripheral vasculature—offer a non‐invasive, real‐time window into tumor dynamics, minimal residual disease (MRD), and therapeutic resistance across various solid tumors [26, 27]. Preoperative CTC enumeration and dynamic clearance have been extensively validated as independent prognostic markers for long‐term survival and systemic relapse [28, 29]. However, while the prognostic utility of CTCs in advanced or metastatic NSCLC is well‐documented, investigations specifically focusing on CTC dynamics to assess pathological response in the neoadjuvant immunotherapy setting are still in their infancy. Recent clinical reports have begun to highlight this potential, demonstrating that a rapid decline or complete clearance of CTCs during neoadjuvant immune checkpoint blockade strongly correlates with the achievement of pCR upon surgical resection [30, 31]. Our study of 39 patients provided supplementary evidence for this emerging paradigm, and our exploratory findings suggested that CTC testing may help resolve the discordance between radiological evaluation and true pathological clearance prior to surgery.

A notable limitation of the current study is the absence of systematic, longitudinal CTC monitoring across the entire patient cohort. Nevertheless, an exploratory evaluation of the six patients with available serial CTC assessments yielded compelling preliminary insights. Within this subset, the dynamic trajectories of CTC clearance or persistence appeared to closely mirror the ultimate pathological response, occasionally resolving the predictive ambiguity of concurrent radiographic changes. By framing it this way, we maintain the clinical interest in the data (i.e., that CTC trajectories mirrored pathological response in that subset) while explicitly acknowledging that the sample size is insufficient for a definitive “validated” claim. Future prospective studies incorporating strictly timed, serial liquid biopsies are warranted to fully validate the kinetic utility of CTCs in the neoadjuvant setting.

A substantial body of existing literature demonstrates that preoperative PD‐L1 expression on CTCs frequently serves as a reliable predictive biomarker for favorable clinical responses to immune checkpoint blockade in NSCLC [29, 32]. However, in the present cohort, CTC PD‐L1 positivity did not exhibit this anticipated predictive potential for pathological clearance. The reason we hypothesized is that the majority of the evaluated CTC specimens in this study were acquired after the initiation of neoadjuvant chemoimmunotherapy, rather than at a strictly treatment‐naïve baseline. The phenotypic profile of disseminated tumor cells is highly dynamic and subject to intense selective pressure during therapy [33]. We hypothesized that in patients who achieved a favorable therapeutic response, the neoadjuvant regimen may have eradicated the PD‐L1‐expressing CTC clones or induced a downregulation of surface PD‐L1 expression prior to the time of blood collection, which may also explain the low spatial concordance between baseline tissue PD‐L1 expression and post‐treatment CTC PD = L1 status. Future prospective studies should incorporate standardized, longitudinal CTC monitoring throughout the treatment course to fully elucidate the dynamic predictive value of CTC PD‐L1 expression in the neoadjuvant immunotherapy setting.

A particularly intriguing exploratory observation in our cohort was the identification of two patients who achieved a confirmed pCR in the surgical specimen yet maintained detectable CTCs in their peripheral blood. This discordance suggested that systemic cellular persistence can occur despite successful local tissue eradication. Although achieving a pCR is traditionally recognized as a robust surrogate endpoint for long‐term survival in NSCLC, the concomitant presence of CTCs may serve as a potential indicator of a latent risk for distant relapse [34]. Trials including CheckMate 816 and NADIM have increasingly highlighted that the failure to clear circulating tumor markers (such as ctDNA) during neoadjuvant therapy remains a strong independent predictor of inferior disease‐free survival, even in patients exhibiting MPR [6, 35]. Given the relatively short follow‐up duration of the current study, it remains to be seen whether the persistent circulating burden in these specific individuals will ultimately translate into overt clinical recurrence. Consequently, our findings highlight the potential value of integrating longitudinal liquid biopsies with standard pathology to identify high‐risk subsets who might benefit from intensified adjuvant surveillance [36].

To elucidate the biological mechanisms underlying differential CTC dissemination, we integrated our liquid biopsy findings with bulk transcriptomic profiling of the residual tumor microenvironment (TME). Our GSEA suggested that systemic CTC dissemination is linked to a microenvironmental convergence of proliferative activity, severe local hypoxia, and comprehensive immunosuppression within the primary tumor bed. Specifically, residual tumors harboring persistent CTCs exhibited a pronounced enrichment of mitotic and cell cycle pathways, together with a significant upregulation of canonical hypoxia‐responsive elements, including *CA9* and *SLC2A1* (GLUT1). Foundational research has established that a highly hypoxic TME can induce metabolic reprogramming and trigger epithelial‐mesenchymal transition (EMT), thereby promoting extracellular matrix degradation and facilitating the intravasation of malignant cells into the peripheral bloodstream [37, 38]. It is biologically plausible that specific tumor sub‐clones, capable of enduring extreme oxygen deprivation and metabolic stress, acquire an enhanced invasive capacity that drives hematogenous dissemination.

In addition to these proliferative and metabolic shifts, our transcriptomic analysis also suggested the potential contribution of an “immune‐cold” microenvironment to tumor cell escape. GSEA demonstrated a widespread attenuation of both cellular and humoral immune networks in the residual tissues of CTC‐positive patients, characterized by the marked suppression of TNF signaling, Th17 cell differentiation, and local immunoglobulin production. Previous studies indicated that a deeply immunosuppressive TME not only blunts the efficacy of primary immune clearance but may also pre‐condition intravasating cells to successfully evade peripheral immune surveillance, allowing them to persist as viable CTCs in the circulation [39, 40]. Consequently, the synergistic interplay between local hypoxia and immune exhaustion may represent a key biological vulnerability that facilitates residual tumor dissemination despite a macroscopic radiographic response.

We acknowledge several important limitations in the present study that warrant consideration. First, this was a single‐center, prospective analysis with a relatively small sample size (*N* = 39) and lacked an independent validation cohort, which limited the statistical power of subgroup analyses and the broader generalizability of our findings. Furthermore, as noted previously, the lack of systematic, longitudinal liquid biopsy collection across the entire cohort precluded a comprehensive evaluation of real‐time CTC dynamics. Consequently, our predictive models require rigorous validation in larger, prospective, multicenter cohorts. Second, the primary endpoint of this study was restricted to pathological response (pCR and MPR). While pathological clearance is a well‐established surrogate for therapeutic efficacy in the neoadjuvant setting, the current follow‐up duration is insufficient to assess long‐term clinical outcomes, such as disease‐free survival (DFS) and overall survival (OS). Therefore, the definitive prognostic impact of preoperative CTC burden on long‐term survival cannot yet be confirmed. Long‐term survival data, including disease‐free survival (DFS) and overall survival (OS), are being systematically collected and will be analyzed and reported in future publications as the data reach maturity. Third, we humbly recognize that the specific clinical composition of our cohort, which is predominantly male and characterized by a high proportion of squamous cell histology, may introduce inherent selection biases that could potentially influence the generalizability of these observed trends. Fourth, we recognize that the exclusive use of CK as an epithelial marker may not capture CK‐negative CTC subpopulations that have undergone epithelial–mesenchymal transition. Future studies incorporating mesenchymal markers such as Vimentin or N‐cadherin could potentially improve detection sensitivity [41]. Finally, our microenvironmental analyses were dependent on bulk RNA‐sequencing. Although this approach successfully captured global transcriptomic shifts, it inherently lacks the single‐cell resolution required to delineate precise cell‐to‐cell interactions or identify the exact cellular origins of these transcriptomic signals. Future investigations utilizing single‐cell RNA sequencing (scRNA‐seq) or spatial transcriptomics, combined with dedicated in vitro and in vivo functional assays, are essential to mechanistically validate the TME‐driven CTC dissemination pathways proposed herein.

## 5 Conclusions

In conclusion, the present exploratory study suggested that CTCs may serve as a promising complementary biomarker to traditional radiographic imaging for patients undergoing neoadjuvant chemoimmunotherapy. By integrating liquid biopsy assessments with transcriptomic profiling of the residual tumor microenvironment, we preliminarily revealed the potential correlation between local treatment resistance and systemic tumor dissemination. Collectively, these macroscopic and microscopic findings provide a novel theoretical foundation for the precise, multidimensional evaluation of preoperative therapeutic efficacy. Ultimately, the incorporation of CTC monitoring into standard clinical workflows holds potential promise for helping resolve imaging ambiguities, refining prognostic stratification, and optimizing individualized treatment strategies, which need further validation in large multicenter cohorts.

## Author Contributions

**Jianyi Yang:** conceptualization, methodology, formal analysis, writing – original draft. **Ziwen Qin:** methodology, investigation, data curation, writing – original draft. **Zhiwei Zhou:** formal analysis, visualization, validation, writing – original draft. **Jixian Liu:** investigation, resources, project administration. **Lin Chen:** investigation, resources, validation. **Ziyan Mo:** formal analysis, software, visualization. **Mengying Liao:** investigation, data curation. **Dongliang Cui:** resources, validation. **Kaiqin Wu:** software, funding acquisition, data curation. **Dongjiang Tang:** methodology, resources. **Jinfeng Chen:** conceptualization, supervision, project administration, writing – review and editing. **Chao Chen:** funding acquisition, conceptualization, supervision, writing – review and editing.

## Funding

This work was supported by Sanming Project of Medicine in Shenzhen (No. SZSM202411027), the Shenzhen Medical Research Fund under Grant No. A2403061, and Shenzhen Science and Technology Program under Grant No. JCYJ20240813120107010.

## Ethics Statement

This study was approved by the Ethics Committee of Peking University Shenzhen Hospital (No. 2020‐074) and all procedures performed in this study were in accordance with the Declaration of Helsinki.

## Consent

Informed consent was obtained from all individual participants.

## Conflicts of Interest

Lin Chen and Dongjiang Tang are current operators of Zhuhai Sanmed Biotech Ltd., and they completed the experimental test detection work for this study. The other authors have no conflicts of interest to declare.

## Supporting information


**Table S1:** tca70288‐sup‐0001‐TableS1.xlsx.

## Data Availability

The data that support the findings of this study are available on request from the corresponding author. The data are not publicly available due to privacy or ethical restrictions.

## References

[1] H. Sung , J. Ferlay , R. L. Siegel , et al., “Global Cancer Statistics 2020: GLOBOCAN Estimates of Incidence and Mortality Worldwide for 36 Cancers in 185 Countries,” CA: A Cancer Journal for Clinicians 71, no. 3 (2021): 209–249. 33538338 10.3322/caac.21660

[2] R. L. Siegel , A. N. Giaquinto , and A. Jemal , “Cancer Statistics, 2024,” CA: A Cancer Journal for Clinicians 74, no. 1 (2024): 12–49. 38230766 10.3322/caac.21820

[3] I. Otano , A. C. Ucero , J. Zugazagoitia , and L. Paz‐Ares , “At the Crossroads of Immunotherapy for Oncogene‐Addicted Subsets of NSCLC,” Nature Reviews. Clinical Oncology 20, no. 3 (2023): 143–159. 10.1038/s41571-022-00718-x 36639452

[4] Preoperative chemotherapy for non‐small‐cell lung cancer , “A Systematic Review and Meta‐Analysis of Individual Participant Data,” Lancet 383, no. 9928 (2014): 1561–1571. 24576776 10.1016/S0140-6736(13)62159-5 PMC4022989

[5] J. D. Spicer , T. Cascone , M. W. Wynes , et al., “Neoadjuvant and Adjuvant Treatments for Early Stage Resectable NSCLC: Consensus Recommendations From the International Association for the Study of Lung Cancer,” Journal of Thoracic Oncology 19, no. 10 (2024): 1373–1414. 38901648 10.1016/j.jtho.2024.06.010

[6] P. M. Forde , J. Spicer , S. Lu , et al., “Neoadjuvant Nivolumab Plus Chemotherapy in Resectable Lung Cancer,” New England Journal of Medicine 386, no. 21 (2022): 1973–1985. 35403841 10.1056/NEJMoa2202170 PMC9844511

[7] J. D. Spicer , M. C. Garassino , H. Wakelee , et al., “Neoadjuvant Pembrolizumab Plus Chemotherapy Followed by Adjuvant Pembrolizumab Compared With Neoadjuvant Chemotherapy Alone in Patients With Early‐Stage Non‐Small‐Cell Lung Cancer (KEYNOTE‐671): A Randomised, Double‐Blind, Placebo‐Controlled, Phase 3 Trial,” Lancet 404, no. 10459 (2024): 1240–1252. 39288781 10.1016/S0140-6736(24)01756-2 PMC11512588

[8] J. Remon , A. Levy , R. Gille , et al., “Unresectable Stage III Non‐Small‐Cell Lung Cancer: State of the Art and Challenges,” Nature Reviews. Clinical Oncology 23, no. 1 (2026): 22–39. 10.1038/s41571-025-01080-4 41068447

[9] H. J. Park , K. W. Kim , J. Pyo , et al., “Incidence of Pseudoprogression During Immune Checkpoint Inhibitor Therapy for Solid Tumors: A Systematic Review and Meta‐Analysis,” Radiology 297, no. 1 (2020): 87–96. 32749204 10.1148/radiol.2020200443 PMC7526949

[10] H. J. Park , K. W. Kim , S. E. Won , et al., “Definition, Incidence, and Challenges for Assessment of Hyperprogressive Disease During Cancer Treatment With Immune Checkpoint Inhibitors: A Systematic Review and Meta‐Analysis,” JAMA Network Open 4, no. 3 (2021): e211136. 33760090 10.1001/jamanetworkopen.2021.1136 PMC7991969

[11] W. Zhang , Z. Liang , Y. Zhao , et al., “Efficacy and Safety of Neoadjuvant Immunotherapy Plus Chemotherapy Followed by Adjuvant Immunotherapy in Resectable Non‐Small Cell Lung Cancer: A Meta‐Analysis of Phase 3 Clinical Trials,” Frontiers in Immunology 15 (2024): 1359302. 38646542 10.3389/fimmu.2024.1359302 PMC11026587

[12] L. E. Cortés‐Hernández , S. Z. Eslami , K. Pantel , and C. Alix‐Panabières , “Circulating Tumor Cells: From Basic to Translational Research,” Clinical Chemistry 70, no. 1 (2024): 81–89. 38175586 10.1093/clinchem/hvad142 PMC10765989

[13] S. H. Hussain , C. S. Huertas , A. Mitchell , A. L. Deman , and E. Laurenceau , “Biosensors for Circulating Tumor Cells (CTCs)‐Biomarker Detection in Lung and Prostate Cancer: Trends and Prospects,” Biosensors & Bioelectronics 197 (2022): 113770. 34768065 10.1016/j.bios.2021.113770

[14] D. de Miguel‐Perez , F. G. Ortega , R. G. Tejada , et al., “Baseline Extracellular Vesicle miRNA‐30c and Autophagic CTCs Predict Chemoradiotherapy Resistance and Outcomes in Patients With Lung Cancer,” Biomarker Research 11, no. 1 (2023): 98. 37968730 10.1186/s40364-023-00544-y PMC10652484

[15] A. C. Kakouri , M. Spiliotaki , E. M. Loizidou , et al., “Monitoring Pembrolizumab Response in Patients With Metastatic Non‐Small Cell Lung Cancer Using Circulating Tumour DNA and Circulating Tumour Cells,” Translational Lung Cancer Research 14, no. 6 (2025): 1945–1960. 40673096 10.21037/tlcr-2024-1095 PMC12261352

[16] M. Dhar , J. Wong , J. Che , et al., “Evaluation of PD‐L1 Expression on Vortex‐Isolated Circulating Tumor Cells in Metastatic Lung Cancer,” Scientific Reports 8, no. 1 (2018): 2592. 29416054 10.1038/s41598-018-19245-w PMC5803213

[17] L. Zhang , X. Zhang , Y. Liu , et al., “PD‐L1(+) Aneuploid Circulating Tumor Endothelial Cells (CTECs) Exhibit Resistance to the Checkpoint Blockade Immunotherapy in Advanced NSCLC Patients,” Cancer Letters 469 (2020): 355–366. 31678168 10.1016/j.canlet.2019.10.041

[18] R. Harouaka , Z. Kang , S. Y. Zheng , and L. Cao , “Circulating Tumor Cells: Advances in Isolation and Analysis, and Challenges for Clinical Applications,” Pharmacology & Therapeutics 141, no. 2 (2014): 209–221. 24134902 10.1016/j.pharmthera.2013.10.004 PMC3947247

[19] V. Kloten , R. Lampignano , T. Krahn , and T. Schlange , “Circulating Tumor Cell PD‐L1 Expression as Biomarker for Therapeutic Efficacy of Immune Checkpoint Inhibition in NSCLC,” Cells 8, no. 8 (2019): 809. 31374957 10.3390/cells8080809 PMC6721635

[20] S. E. Won , H. J. Park , S. Byun , et al., “Impact of Pseudoprogression and Treatment Beyond Progression on Outcome in Patients With Non‐Small Cell Lung Cancer Treated With Immune Checkpoint Inhibitors,” Oncoimmunology 9, no. 1 (2020): 1776058. 32923136 10.1080/2162402X.2020.1776058 PMC7458612

[21] F. Mulkey , M. R. Theoret , P. Keegan , R. Pazdur , and R. Sridhara , “Comparison of iRECIST Versus RECIST V.1.1 in Patients Treated With an Anti‐PD‐1 or PD‐L1 Antibody: Pooled FDA Analysis,” Journal for Immunotherapy of Cancer 8, no. 1 (2020): e000146. 32107275 10.1136/jitc-2019-000146 PMC7057528

[22] H. J. Park , G. H. Kim , K. W. Kim , et al., “Comparison of RECIST 1.1 and iRECIST in Patients Treated With Immune Checkpoint Inhibitors: A Systematic Review and Meta‐Analysis,” Cancers (Basel) 13, no. 1 (2021): 120. 33561078 10.3390/cancers13010120 PMC7795764

[23] H. Chen , B. Fan , M. Yuan , et al., “CT‐Based Radiomics in Predicting the Efficacy of Preoperative Neoadjuvant Chemoimmunotherapy for Non‐Small Cell Lung Cancer: A Systematic Review and Meta‐Analysis,” Frontiers in Immunology 17 (2026): 1753166. 41743740 10.3389/fimmu.2026.1753166 PMC12929539

[24] J. Zheng , Z. Yan , R. Wang , et al., “NeoPred: Dual‐Phase CT AI Forecasts Pathologic Response to Neoadjuvant Chemo‐Immunotherapy in NSCLC,” Journal for Immunotherapy of Cancer 13, no. 5 (2025): e011773. 40449955 10.1136/jitc-2025-011773 PMC12163334

[25] Z. Y. Chen , R. Fu , X. Y. Tan , et al., “Dynamic (18) F‐FDG PET/CT Can Predict the Major Pathological Response to Neoadjuvant Immunotherapy in Non‐Small Cell Lung Cancer,” Thorac Cancer 13, no. 17 (2022): 2524–2531. 35822254 10.1111/1759-7714.14562 PMC9436661

[26] M. Capuozzo , F. Ferrara , M. Santorsola , A. Zovi , and A. Ottaiano , “Circulating Tumor Cells as Predictive and Prognostic Biomarkers in Solid Tumors,” Cells 12, no. 22 (2023): 2590. 37998325 10.3390/cells12222590 PMC10670669

[27] C. Alix‐Panabières and K. Pantel , “From Discovery to Diagnosis: A Perspective for Circulating Tumor Cells in Personalized Oncology,” Cancer Discovery 15, no. 10 (2025): 1985–2001. 40825223 10.1158/2159-8290.CD-25-0164

[28] F. Kakizaki , K. Oshiro , Y. Enoki , et al., “Precision Oncology Framework Using Circulating Tumor Cells,” International Journal of Molecular Sciences 26, no. 12 (2025): 5539. 40565003 10.3390/ijms26125539 PMC12192747

[29] L. Chen , Z. Yang , Y. Lu , S. Li , D. Tang , and L. Zhang , “Analyzed PD‐L1‐Positive Subpopulations by Dual‐Labeling TSA‐IF‐FISH Predicts Immunotherapy Efficacy in Advanced Lung Cancer,” iScience 29, no. 1 (2026): 114357. 41541685 10.1016/j.isci.2025.114357 PMC12799786

[30] W. F. Tang , W. Xu , W. Z. Huang , et al., “Pathologic Complete Response After Neoadjuvant Tislelizumab and Chemotherapy for Pancoast Tumor: A Case Report,” Thoric Cancer 12, no. 8 (2021): 1256–1259. 10.1111/1759-7714.13910 PMC8046127 33656285

[31] Y. Wang , X. Yang , X. Tian , et al., “Neoadjuvant Immunotherapy Plus Chemotherapy Achieved Pathologic Complete Response in Stage IIIB Lung Adenocarcinoma Harbored EGFR G779F: A Case Report,” Annals of Palliative Medicine 9, no. 6 (2020): 4339–4345. 33183013 10.21037/apm-20-1692

[32] N. Guibert , M. Delaunay , A. Lusque , et al., “PD‐L1 Expression in Circulating Tumor Cells of Advanced Non‐Small Cell Lung Cancer Patients Treated With Nivolumab,” Lung Cancer 120 (2018): 108–112. 29748004 10.1016/j.lungcan.2018.04.001

[33] J. A. Moran , D. L. Adams , M. J. Edelman , et al., “Monitoring PD‐L1 Expression on Circulating Tumor‐Associated Cells in Recurrent Metastatic Non‐Small‐Cell Lung Carcinoma Predicts Response to Immunotherapy With Radiation Therapy,” JCO Precision Oncology 6 (2022): e2200457. 36516370 10.1200/PO.22.00457 PMC10166406

[34] J. Tie , J. D. Cohen , K. Lahouel , et al., “Circulating Tumor DNA Analysis Guiding Adjuvant Therapy in Stage II Colon Cancer,” New England Journal of Medicine 386, no. 24 (2022): 2261–2272. 35657320 10.1056/NEJMoa2200075 PMC9701133

[35] M. Provencio , E. Nadal , A. Insa , et al., “Neoadjuvant Chemotherapy and Nivolumab in Resectable Non‐Small‐Cell Lung Cancer (NADIM): An Open‐Label, Multicentre, Single‐Arm, Phase 2 Trial,” Lancet Oncology 21, no. 11 (2020): 1413–1422. 32979984 10.1016/S1470-2045(20)30453-8

[36] R. S. Herbst , T. John , C. Grohé , et al., “Molecular Residual Disease Analysis of Adjuvant Osimertinib in Resected EGFR‐Mutated Stage IB‐IIIA Non‐Small‐Cell Lung Cancer,” Nature Medicine 31, no. 6 (2025): 1958–1968. 10.1038/s41591-025-03577-y PMC12176615 40097663

[37] I. Godet , H. H. Oza , Y. Shi , et al., “Hypoxia Induces ROS‐Resistant Memory Upon Reoxygenation In Vivo Promoting Metastasis in Part via MUC1‐C,” Nature Communications 15, no. 1 (2024): 8416. 10.1038/s41467-024-51995-2 PMC11438863 39341835

[38] E. B. Rankin and A. J. Giaccia , “Hypoxic Control of Metastasis,” Science 352, no. 6282 (2016): 175–180. 27124451 10.1126/science.aaf4405 PMC4898055

[39] K. Pantel and C. Alix‐Panabières , “Crucial Roles of Circulating Tumor Cells in the Metastatic Cascade and Tumor Immune Escape: Biology and Clinical Translation,” Journal for Immunotherapy of Cancer 10, no. 12 (2022): e005615. 36517082 10.1136/jitc-2022-005615 PMC9756199

[40] Y. Sun , J. Zhou , J. Fan , and X. Yang , “Single‐Cell RNA Sequencing Reveals Spatial Heterogeneity and Immune Evasion of Circulating Tumor Cells,” Cancer Biology & Medicine 18, no. 4 (2021): 934–936. 35959966 10.20892/j.issn.2095-3941.2021.0466 PMC8610158

[41] X. Xie , L. Wang , X. Wang , et al., “Evaluation of Cell Surface Vimentin Positive Circulating Tumor Cells as a Diagnostic Biomarker for Lung Cancer,” Frontiers in Oncology 11 (2021): 672687. 34055642 10.3389/fonc.2021.672687 PMC8162210

